# Validation of Hemoglobin and Hematocrit Measurements from a Dialysis Machine Sensor Compared to Laboratory Analysis

**DOI:** 10.3390/jcm14155242

**Published:** 2025-07-24

**Authors:** Niccolò Morisi, Marco Ferrarini, Laura Veronesi, Giovanni Manzini, Silvia Giovanella, Gaetano Alfano, Lucia Stipo, Fabio Olmeda, Giulia Ligabue, Grazia Maria Virzì, Valentina Di Pinto, Luigi Rovati, Gabriele Donati

**Affiliations:** 1 Surgical, Medical, Dental and Morphological Sciences Department (CHIMOMO), University of Modena and Reggio Emilia, 41124 Modena, Italy; marco.ferrarini@unimore.it (M.F.); 253752@studenti.unimore.it (G.M.); gabriele.donati@unimore.it (G.D.); 2 Nephrology, Dialysis and Renal Transplant Unit, Azienda Ospedaliero Universitaria di Modena, 41124 Modena, Italygiulia.ligabue@unimore.it (G.L.); 3 IRRIV—International Renal Research Institute Vicenza-Foundation, 36100 Vicenza, Italy; 4 Department of Nephrology, Dialysis and Transplantation, San Bortolo Hospital, 36100 Vicenza, Italy; 5 Laboratory of Optoelectronics, Department of Engineering “Enzo Ferrari” (DIEF), University of Modena and Reggio Emilia, 41124 Modena, Italy; valentina.dipinto@unimore.it (V.D.P.);

**Keywords:** hemodialysis, hemoglobin, hematocrit, blood volume monitoring, sensor validation

## Abstract

**Background:** Continuous monitoring of hemoglobin (HB) and hematocrit (HCT) during hemodialysis could improve fluid management and patient safety. The Fresenius 5008 dialysis machine includes an ultrasound-based sensor that estimates HB and HCT values, though its accuracy compared to standard laboratory measurements remains unclear. **Methods:** This exploratory observational study assessed the agreement between sensor-derived and laboratory-derived HB and HCT values in 20 patients at the start of hemodiafiltration. A total of 177 paired blood samples were collected. **Results:** Sensor values significantly underestimated laboratory HB (9.61 vs. 11.31 g/dL) and HCT (27% vs. 34%) (*p* < 8 × 10^−25^). Correlations were strong for both parameters (HB: r = 0.788; HCT: r = 0.876). Regression analyses revealed consistent proportional bias. Applying a fixed correction of +1.69 g/dL for HB and +7.55% for HCT eliminated the statistical differences and reduced intercepts in regression models. Bland–Altman plots confirmed improved agreement post-correction. Albumin levels correlated modestly with error magnitude. **Conclusions:** HB and HCT values from the Fresenius 5008 sensor are strongly correlated with laboratory data but are systematically underestimated at treatment start, likely due to hemodilution. Applying fixed correction factors improves accuracy and supports the sensor’s use for real-time monitoring.

## 1. Introduction

Effective monitoring of fluid removal remains a major challenge in the management of patients undergoing dialysis. As a direct measurement of blood volume is not feasible, recent technological advances have enabled the integration of indirect, non-invasive methods into modern dialysis machines. These techniques allow for continuous monitoring of blood volume changes and hemoconcentration by tracking dynamic fluctuations of HB or protein concentrations at the arterial port, using tools such as optical photometry or ultrasonic measurement.

All current methods are based on the principle that blood elements, such as plasma proteins or cells, remain within the vascular space. As plasma water is removed through ultrafiltration, the concentration of these components increases during the treatment process. Various manufacturers have developed systems based on this principle, differing in both the measured blood components and the techniques employed [1,2,3,4,5,6].

One such device, the Blood Volume Monitor (BVM, Fresenius AG, Bad Homburg, Germany), integrated into the Fresenius 5008 HD machine, calculates relative blood volume (ΔRBV) by measuring total protein concentration (TPC)—the combined concentration of plasma proteins and HB—using an ultrasonic method. This technique measures the sound velocity in blood by recording the transit time of acoustic pulses in the arterial line. A silicon rubber insert ensures proper sound coupling, while temperature corrections are applied via a high-precision sensor. TPC is derived from sound velocity and temperature through an empirical function, and ΔRBV is then determined as a function of TPC over time [7]. HCT and HB concentrations are estimated through linear equations, assuming a standard plasma protein concentration of 72.5 g/L at baseline [8].

The continuous assessment of hemoconcentration has not only facilitated a more precise control of optimal fluid status, avoiding long-term fluid overload, a known contributing factor to cardiac stress, but has also contributed to improving patient tolerance during dialysis [9]. Specifically, real-time monitoring of hemoconcentration enables the early detection of rapid blood volume reductions, thereby allowing for prompt intervention to prevent ultrafiltration-induced hypovolemia and hypotension—a common and potentially life-threatening complications of dialysis, with an estimated prevalence of 11.6%, according to a recent meta-analysis [10].

Despite widespread clinical use of blood volume monitoring, its potential for real-time HB and HCT assessment remains underexplored. The implementation of a continuous measurement of these parameters could offer valuable insights into patient status while reducing the need for additional blood sampling. In particular, routine HB monitoring during dialysis sessions may lead to further refinement of anemia management, allowing early detection of anemia, and reducing the response time for appropriate clinical therapeutic action.

This study aims to validate the accuracy of HB and HCT measurements provided by the 5008 CorDiax (Fresenius, Mumbai, Maharashtra) dialysis machine in a population undergoing chronic intermittent hemodiafiltration. The investigation will assess whether continuous hemoconcentration monitoring can reliably reflect the hematological status of patients in comparison to standard hemochrome laboratory analysis. This will determine whether technology has the potential to serve as a dual-purpose tool, both for optimizing fluid management and for providing real-time, actionable data on HB and HCT levels, thus contributing to a more refined and responsive dialysis therapy.

## 2. Materials and Methods

### 2.1. Study Design

This is an ancillary study of the CHORALS protocol (Comparison of two different dialyzers for online Hemodiafiltration by Toxins Removal and Inflammatory State modulation). This study is a validation study used to assess the diagnostic accuracy of hemochrome values (HB and HCT) obtained by a dialysis machine and the conventional hemochrome obtained by laboratory analysis. Using an exploratory observational study, we collected samples according to protocol. The analysis was performed from 14 February 2024 to 7 March 2025. The study was conducted in the Hemodialysis center of Policlinico of Modena (MO).

### 2.2. Audit Population

As part of a CHORALS protocol and clinical practice, blood samples were collected from patients during high flux online hemodiafiltration (HDF, Singapore). The samples were obtained before the commencement of the HDF session from the vascular access. Following the CHORALS protocol, all patients were treated with a 5008 Cordiax machine (Fresenius Medical Care, Bad Homburg, Germany). HB and HCT values were obtained by the monitor at the beginning of each HDF session. Each patient contributed to multiple dialysis sessions, with a total of 177 paired blood samples obtained across routine mid-week treatments. The number of samples per patient ranged from 6 to 10, depending on clinical availability and adherence to the study period. Blood draws were performed at the start of each dialysis session, with both sensor readings and corresponding laboratory samples collected simultaneously from the arterial line. In a subset of 15 treatment sessions, a second paired sample was collected one hour after treatment initiation, allowing for an exploratory mid-session comparison.

Among the 20 patients included in the study, 12 were male (60%). The mean age was 60.9 ± 16 years with a mean HD vintage of 58.6 ± 51 months. The mean body mass index (BMI) was 24.9 ± 3.2 Kg/m^2^. The majority of patients were Caucasian (70%), followed by a smaller proportion of Africans (25%), and just 1 patient was Asian (5%). The causes of ESKD were well distributed between primary glomerulopathy (25%), urological (25%), secondary nephropathy to cardiovascular disease (20%), genetic cause (15%), unknown (10%), and autoimmune disease (5%). Hypertension was the most common comorbidity observed (65%), followed by diabetes (20%), heart failure (15%), chronic infections (10%), and past stroke (5%).

### 2.3. Clinical Specimens

All patients underwent treatment according to our standardized clinical protocol. Laboratory HB and HCT were obtained by DxH 900 hematology analyzer (Beckman Coulter Inc., Brea, CA, USA). The measurement of HB and HCT in the dialysis machine was facilitated by a dedicated cuvette positioned within the arterial tubing system. The cuvette determined the blood density using the ultrasound system. Following the calibration of the cuvette during the preparation of the dialysis machine, as verified by the monitor, the sensor determined the values of HB and HCT as an indirect measure. In order to explore potential biochemical confounders, plasma albumin concentration was also analyzed, using results from routine laboratory tests performed on the same day as the dialysis session.

### 2.4. Statistical Analysis

All statistical analyses were conducted using R (version 4.2.0). The normality of continuous variables was assessed using the Shapiro–Wilk and Kolmogorov–Smirnov tests, depending on the sample size. For descriptive purposes, results are expressed as mean ± standard deviation (SD) for normally distributed variables, and as median and interquartile range (IQR) for non-normally distributed ones. Comparisons between the values measured by the Fresenius 5008 dialysis machine and the corresponding laboratory values (for HB and HCT) were performed using appropriate parametric or non-parametric tests, depending on the distribution of the data. Analyses were conducted both on absolute values and on the delta values, defined as the variation between the beginning and the end of the dialysis session. Agreement between the two measurement methods was assessed using the Bland–Altman analysis, which allowed for the identification of any systematic bias and the calculation of the limits of agreement. The Pearson (r) or Spearman correlation coefficient (based on data distribution) was used to evaluate the strength and direction of the relationship between the machine-derived and laboratory-derived values. In addition, we examined whether a linear correction factor could be applied to the machine-derived values to minimize the difference between the two methods. To this end, linear regression analysis was performed, and the corrected values were re-evaluated for agreement and error reduction. Linear regression analysis was performed to evaluate the relationship between sensor-derived and laboratory-derived values for both HB and HCT. In each model, the laboratory value was treated as the dependent variable (Y), and the value measured by the Fresenius 5008 sensor as the independent variable (X).

The regression model included estimation of slope (β), intercept, and the coefficient of determination (R^2^). Ninety-five percent confidence intervals (95% CI) were calculated for both the slope and the intercept. Statistical significance was assessed using the F-test for overall model fit.

Additionally, we used the derived regression equations to define correction factors, which were then applied to sensor values. The corrected values were re-analyzed using the same statistical methods to verify the reduction in bias and improvement in agreement with laboratory data.

A *p*-value < 0.05 was considered statistically significant.

### 2.5. Endpoints

The primary endpoint of this study is to evaluate the accuracy of hemoglobin (HB) and hematocrit (HCT) values measured by the Fresenius 5008 ultrasound-based sensor compared to the corresponding values obtained through standard laboratory analysis, which is considered the reference method. As a secondary endpoint, we aim to assess the potential influence of serum protein concentration on the discrepancy between the two measurement methods. In addition, we investigate whether a correction factor can be identified and applied to the device-generated values in order to improve agreement with laboratory measurements.

## 3. Results

A total of 177 blood samples were collected at the start of dialysis sessions for analysis. The baseline values for laboratory hematological measurements were as follows: mean HCT 34% (SD 3.504%) and mean HB 11.31 g/dL (SD 1.042 g/dL). Corresponding measurements obtained by the dialysis sensor showed a mean HCT of 27% (SD 3.387%) and a mean HB of 9.61 g/dL (SD 0.980 g/dL). Normal distribution was confirmed for all variables. See Table A1 in Appendix A for more details.

### 3.1. Hemoglobin and Hematocrit Measurements

Pearson’s correlation analysis demonstrated a strong correlation between sensor-derived and laboratory-derived values, both for HCT (r = 0.876, *p*-value: 1.20 × 10^−57^) and HB (r = 0.788, *p*-value: 5.95 × 10^−39^). However, the paired *t*-test detected significant differences between the two methods for both parameters (*p* < 8 × 10^−25^).

Subsequently, a linear regression analysis was performed. For HB (*R*^2^ = 0.77, *p* < 2.2 × 10^−16^), the regression equation was as follows:

HB_LAB_ = 0.93HB_SENSOR_ + 2.35 (1)

HB_SENSOR_ = 0.82HB_LAB_ + 0.29 (2)

The model showed a slope of 0.93 (95% CI: 0.855 to 1.00; SE = 0.0387), indicating a nearly proportional relationship. However, the intercept was 2.35 g/dL (95% CI: 1.610 to 3.08; SE = 0.374), suggesting a systematic underestimation by the sensor.

A similar analysis for HCT yielded the following regression equation (*R*^2^ = 0.62, *p* < 2.2 × 10^−16^):

HCT_LAB_ = 0.81HCT_SENSOR_ + 12.6 (3)

HCT_SENSOR_ = 0.76HCT_LAB_ + 0.76 (4)

The regression model yielded a slope of 0.81 (95% CI: 0.719 to 0.900; SE = 0.048) and an intercept of 12.6% (95% CI: 9.99 to 15.22; SE = 1.325), again confirming consistent underestimation of laboratory values by the sensor.

In light of these findings, a constant correction was applied based on the mean differences between methods: 7.55% was added to sensor HCT and 1.69 g/dL was added to sensor HB. After correction, the paired *t*-test no longer detected significant differences for either HCT or HB (*p* > 0.05). Furthermore, regression intercepts were markedly reduced.

For corrected HB, the regression is as follows:

HB_LAB_ = 0.93HB_SENSOR-CORRECTED_ + 0.71 (5)

For corrected HCT, the regression is as follows:

HCT_LAB_ = 0.81HCT_SENSOR-CORRECTED_ + 6.46 (6)

**Figure 1:**
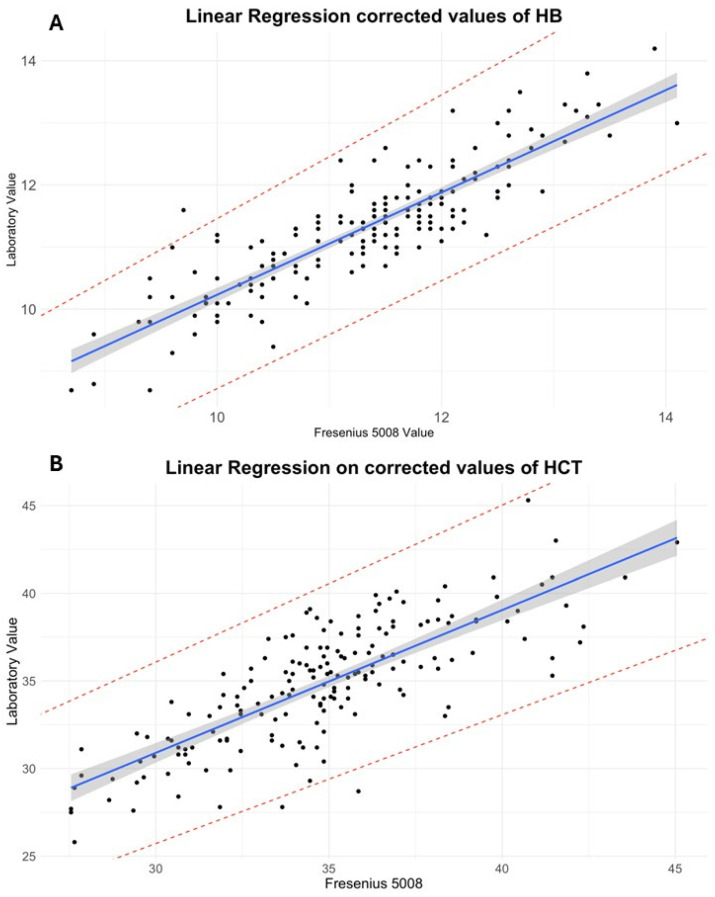
Linear regression: The graphs (**A**,**B**) display the linear regression of hemoglobin (HB, on the upside) and hematocrit (HCT, on the downside); values are corrected using the proposed adjustment factor. Each black dot represents a paired data point (monitor vs. laboratory values), showing a clear linear distribution. The line indicates the regression line, while the shaded gray area represents the 95% confidence interval. Dashed lines mark the 95% prediction limits. HB values are expressed in g/dL, HCT values are expressed in %. The x-axis represents HB or HCT measured using the dialysis sensor, while the y-axis shows the values measured by the laboratory.

See Figure 1A,B for details.

**Figure 2:**
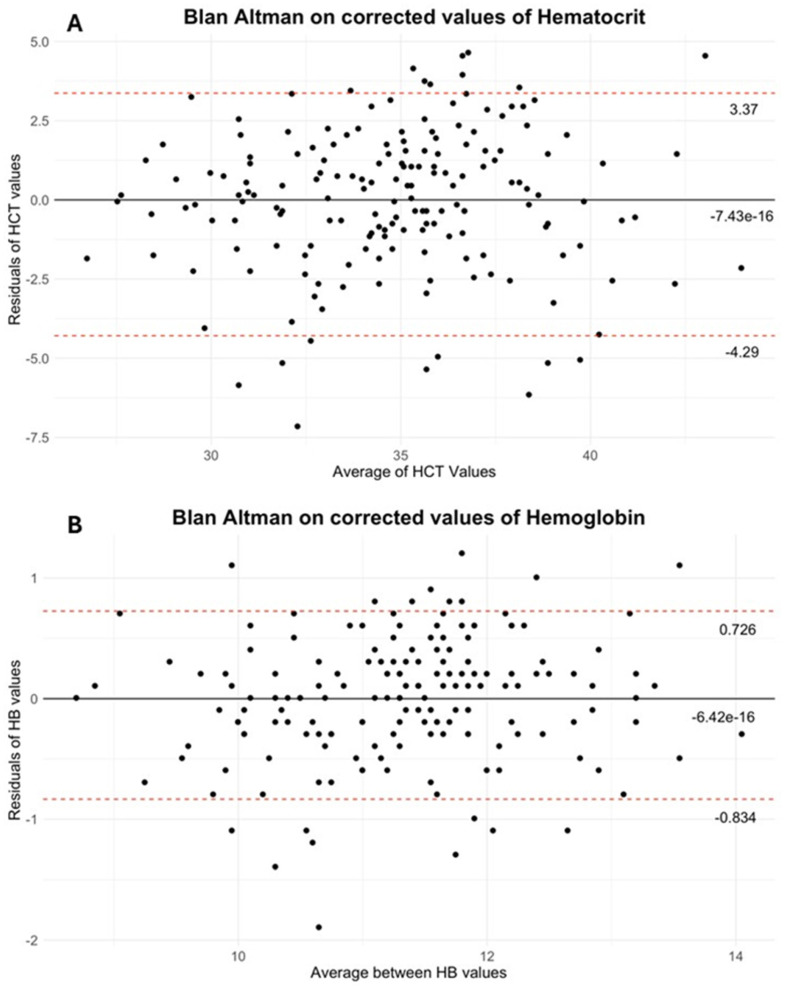
Bland–Altman graphs: The graphs (**A**,**B**) present the Bland–Altman plots (hematocrit (HCT) on the upside and hemoglobin (HB) on the downside) for values that have been corrected using the adjustment factor. As delineated in the paper, the adjustments are validated by the mean difference being near to zero (continued line) and the majority of plots being between the 95% confidence interval (discontinued line).

Finally, the Bland–Altman analysis confirmed that correction substantially reduced the mean bias, resulting in a mean difference of −6.42 × 10^−16^ g/dL for HB (95% limits of agreement: 0.726, −0.834) and −7.43 × 10^−16^% for HCT (95% limits of agreement: 3.37, −4.29). (See Figure 2A,B for Bland–Altman plots).

In a subset of 15 sessions, hemoconcentration after 1 h of dialysis was also assessed using both the sensor and laboratory hemogram values. The sensor-derived mean hemoconcentration was 30.78% (SD 3.337) for HCT and 10.62 g/dL (SD 0.937) for HB, whereas the laboratory-determined mean hemoconcentration was 35.1% (SD 3.22%) for HCT and 11.49 g/dL (SD 1.006 g/dL) for HB. The mean difference between sensor and laboratory values was 4.32% for HCT, with a Pearson correlation of 0.874. For HB, the mean difference was 0.86 g/dL, with a Pearson correlation of 0.916. See Table A2 in Appendix A for more details. Due to the limited sample size of the 1 h subgroup (n = 15), only descriptive analysis and correlation testing were performed. Paired *t*-tests and regression models were not applied, as the statistical power was insufficient to generate robust estimates. This limitation is discussed further in the appropriate section.

### 3.2. Measurement Artifacts

To identify potential confounding factors, plasma albumin (Al) levels were measured at each sampling point. The mean albumin concentration was 3.629 g/dL (SD 0.318). Pearson correlation analysis revealed a correlation between albumin levels and the Sensor-to-Laboratory measurement error of −0.3355 (*p* = 4.965 × 10^−6^, 95% interval: −0.460, −0.198) for HCT and −0.1577 (*p* = 0.036, 95% interval: −0.2983, −0.0104) for HB.

## 4. Discussion

The precise evaluation of HB and HCT levels during the process of hemodialysis is of paramount clinical significance. These parameters are routinely used to inform decisions regarding erythropoiesis-stimulating agent (ESA) therapy, to monitor for excessive hemoconcentration, which may compromise circuit integrity, and to detect potentially life-threatening conditions such as severe anemia or occult blood loss, which may require urgent transfusion [11,12,13]. In recent years, there has been a growing interest in the possibility of continuously monitoring these values in real time through dialysis machine sensors [9,14]. This is due to the potential to improve clinical management and patient safety. In this study, the ability of the integrated sensor in the Fresenius 5008 dialysis system to accurately measure HB and HCT was evaluated, with standard laboratory analysis serving as the reference method. The use of a hemodialysis machine with a reliable sensor for HB and HCT could also be of great practical importance to upgrade the technology behind HB and HCT detection in Continuous Renal Replacement Therapy machines used for AKI patients [15].

Data from the HB regression suggests that 77% of the variance predicted by the model explains the relation between HB determined by the two methods. The slope of the regression line (β = 0.93, 95% CI: 0.855 to 1.00, SE: 0.0387) suggests a near-equal relationship, whereas the intercept (2.35, 95% CI: 1.610 to 3.08, SE: 0.374) indicated a systematic bias, with sensor values tending to underestimate laboratory measurements. For HCT regression, the R^2^ showed that just the slope (β = 0.81, 95% CI: 0.719 to 0.90, SE: 0.048) and intercept (12.6, 95% CI: 9.99 to 15.22, SE: 1.325) confirmed a systematic underestimation by the sensor measurements (see Figure 1 and Appendix A for regression details).

Previous accuracy studies have reported that the Blood Volume Monitor (BVM) estimates HCT with a margin of error of approximately ±2.9% and HB with an error of ±0.8 g/dL. However, these measurements rely on the assumption of a constant plasma protein concentration, and the sensor cannot distinguish between plasma and intracellular proteins. As a result, deviations from the expected protein concentration range (65–80 g/L) may introduce measurement variability and lead to clinically relevant inaccuracies, particularly in patients with hypo- or hyperproteinemia [10,16]. Furthermore, the BVM often modulates ultrafiltration in real time, which increases the clinical impact of any misreading potentially contributing to intradialytic complications such as hypotension or insufficient volume removal [17]. One possible explanation for the systematic bias observed at the beginning of treatment lies in the pre-filling of the extracorporeal circuit with a water-based priming solution. This may transiently dilute the first blood samples analyzed by the sensor, leading to an underestimation of true HB and HCT levels. Although this dilution effect tends to decrease as dialysis progresses, it does not disappear entirely, suggesting the need for a time-sensitive or phase-specific correction.

In the subgroup analysis conducted one hour after dialysis initiation, the discrepancy between sensor and laboratory values was markedly reduced compared to the baseline measurements. This observation likely reflects the progressive equilibration between blood and priming solution over time, which mitigates the initial hemodilution artifact observed at treatment start. This finding suggests that a time-dependent bias may be present and that static correction factors, although appropriate at baseline, may not be generalizable throughout the entire dialysis session.

The reduced sample size in this subset (n = 15) may limit the generalizability of these findings; however, the trend is consistent with a dilutional effect decaying over time. Future research could explore dynamic correction models, potentially incorporating treatment time or sensor-derived blood volume data as covariates to better calibrate measurements across the entire session.

Pearson correlation analysis revealed a statistically significant, though modest, inverse correlation between albumin levels and the measurement error of the sensor compared to laboratory values: r = −0.3355 (*p* < 0.00001) for HCT and r = −0.1577 (*p* = 0.036) for HB. Although these correlations indicate a potential influence of plasma protein concentration on measurement accuracy, the strength of association is limited, particularly for HB.

These results suggest that albumin alone may not be a sufficient explanatory variable, but rather a component of a more complex physiological framework affecting sensor performance. A multivariate correction model, potentially incorporating total plasma proteins alongside albumin, HCT, and treatment time, could better account for interindividual variability in blood composition and improve the predictive accuracy of sensor-derived values.

Future studies with larger datasets should explore this possibility, as well as the potential role of biochemical and hemodynamic parameters as covariates in real-time calibration algorithms.

Based on our results, we propose the application of a fixed correction factor at the start of treatment—+7.55% for HCT and +1.69 g/dL for HB—in order to align sensor-derived values with laboratory measurements. In order to verify the efficacy of the implemented correction, the Bland–Altman method was employed to analyze the data. The results, as illustrated in Figure 2, demonstrate that the mean differences for both HB and HCT are in close proximity to zero.

### Limitations of the Study

This study presents several limitations that should be acknowledged. First, although a relatively large number of blood samples (n = 177) were analyzed, the total patient cohort consisted of only 20 individuals, making this an exploratory study. The limited sample size reduces statistical power and prevents robust subgroup analysis. Second, there was considerable heterogeneity in patient characteristics. The age distribution (60.9 ± 16 years) and hemodialysis vintage (58.6 ± 51 months) show high variability, which may confound interpretation of trends and sensor accuracy. Third, while the cohort was predominantly Caucasian (70%) and hypertensive, the ethnic and comorbidity composition may limit generalizability to broader, more diverse populations. Similarly, the uneven distribution of ESKD etiologies (e.g., diabetic nephropathy, glomerulonephritis) reflects the heterogeneity typically seen in real-world settings, but makes it difficult to isolate the effects of specific clinical variables on measurement accuracy. Fourth, measurements were performed at treatment start, with limited evaluation at 1 h and no data for the final phase of dialysis, thereby limiting insight into sensor performance throughout the full treatment cycle. Lastly, due to the exploratory nature of the study, correction factors were derived empirically and require validation in a larger, prospective cohort before clinical application.

## 5. Conclusions

HB and HCT values measured by the Fresenius 5008 CorDiax BVM at the beginning of dialysis treatment are not consistent with those obtained through standard laboratory analysis, unless a correction is applied. This underestimation of HB and HCT appears systematic and reproducible across patients, enabling the derivation of constant correction factors. Based on our findings, we recommend applying a fixed correction of +7.55% for HCT and +1.69 g/dL for HB to sensor-derived values at treatment start. However, since this dilutional error decreases as treatment progresses, a time-dependent correction model may be warranted for more accurate tracking throughout the dialysis session. Notably, in baseline measurements, laboratory values consistently and significantly exceeded BVM readings—an aspect that should be carefully considered in clinical decision-making.

## Data Availability

The original contributions presented in this study are included in the article. Further inquiries can be directed to the corresponding author.

## Appendix A

**Table A1:** Statistical evaluation of 177 samples collected at the start of the treatment of both hemoglobin (HB) and hematocrit (HCT) dataset before and after correction.

	HB	HCT	HB Corrected	HCT Corrected
Normality assessment LAB	2.639186 × 10^−1^	4.709445 × 10^−1^	2.639186 × 10^−1^	4.709445 × 10^−1^
Normality assessment 5008	9.488492 × 10^−2^	9.281269 × 10^−2^	9.488492 × 10^−2^	9.281269 × 10^−2^
Standard Deviation LAB	1.042314	3.504042	1.042314	3.504042
Standard Deviation 5008	9.800544 × 10^−1^	3.387468	9.800544 × 10^−1^	3.387468
Paired t.test	1.280691 × 10^−97^	5.643302 × 10^−98^	1.000000	1.000000
Paired wilcox test	8.205402 × 10^−31^	8.542299 × 10^−31^	2.940370 × 10^−1^	6.455335 × 10^−1^
mean difference	1.693785	7.550847	−6.422783 × 10^−16^	−7.427863 × 10^−16^
Pearson correlation	8.761294 × 10^−1^	7.876371 × 10^−1^	8.761294 × 10^−1^	7.876371 × 10^−1^
Regression Intercept	2.349787	1.260807 × 10^1^	7.715395 × 10^−1^	6.456078
Regression Slope	9.317870 × 10^−1^	8.147422 × 10^−1^	9.317870 × 10^−1^	8.147422 × 10^−1^
Regression Rsquared	7.676028 × 10^−1^	6.203721 × 10^−1^	7.676028 × 10^−1^	6.203721 × 10^−1^

**Table A2:** Statistical evaluation of 15 samples collected at the first hour of the treatment of both hemoglobin (HB) and hematocrit (HCT) dataset before and after correction.

	HB	HCT	HB Corrected	HCT Corrected
Normality assessment LAB	8.206904 × 10^−1^	7.247844 × 10^−1^	8.206904 × 10^−1^	7.247844 × 10^−1^
Normality assessment 5008	2.621123 × 10^−1^	2.862292 × 10^−1^	2.621123 × 10^−1^	2.862292 × 10^−1^
Standard Deviation LAB	1.006029	3.221136	1.006029	3.221136
Standard Deviation 5008	9.376770 × 10^−1^	3.337707	9.376770 × 10^−1^	3.337707
paired t.test	8.531317 × 10^−7^	7.494155 × 10^−8^	1.000000	1.000000
paired wilcox test	7.157647 × 10^−4^	6.103516 × 10^−5^	9.773172 × 10^−1^	1.000000
mean difference	8.666667 × 10^−1^	4.320000	−4.736979 × 10^−16^	−2.368367 × 10^−16^
Pearson correlation	9.164085 × 10^−1^	8.749849 × 10^−1^	9.164085 × 10^−1^	8.749849 × 10^−1^
Regression Intercept	1.045082	9.108579	1.929665 × 10^−1^	5.460660
Regression Slope	9.832106 × 10^−1^	8.444256 × 10^−1^	9.832106 × 10^−1^	8.444256 × 10^−1^
Regression Rsquared	8.398045 × 10^−1^	7.655986 × 10^−1^	8.398045 × 10^−1^	7.655986 × 10^−1^
